# Development of the Correction Algorithm to Limit the Deformation of Bacterial Colonies Diffraction Patterns Caused by Misalignment and Its Impact on the Bacteria Identification in the Proposed Optical Biosensor

**DOI:** 10.3390/s20205797

**Published:** 2020-10-13

**Authors:** Igor Buzalewicz, Agnieszka Suchwałko, Magdalena Karwańska, Alina Wieliczko, Halina Podbielska

**Affiliations:** 1 Bio-Optics Group, Department of Biomedical Engineering, Faculty of Fundamental Problems of Technology, Wrocław University of Science and Technology, 27 Wybrzeże S. Wyspiańskiego St., 50-370 Wrocław, Poland; halina.podbielska@pwr.edu.pl; 2 QUANTUP, 30/9 Litewska St., 51-354 Wrocław, Poland; agnieszka@quantup.pl; 3 Department of Epizootiology and Veterinary Administration with Clinic of Infectious Diseases, Faculty of Veterinary Medicine, Wrocław University of Environmental and Life Science, 45 Grunwaldzki Square, 50-366 Wrocław, Poland; magdalena.karwanska@upwr.edu.pl (M.K.); alina.wieliczko@upwr.edu.pl (A.W.)

**Keywords:** optical biosensors, bacteria identification, misalignment

## Abstract

Recently proposed methods of bacteria identification in optical biosensors based on the phenomenon of light diffraction on macro-colonies offer over 98% classification accuracy. However, such high accuracy relies on the comparable and repeatable spatial intensity distribution of diffraction patterns. Therefore, it is essential to eliminate all non-species/strain-dependent factors affecting the diffraction patterns. In this study, the impact of the bacterial colony and illuminating beam misalignment on the variation of classification features extracted from diffraction patterns was examined. It was demonstrated that misalignment introduced by the scanning module significantly affected diffraction patterns and extracted classification features used for bacteria identification. Therefore, it is a crucial system-dependent factor limiting the identification accuracy. The acceptable misalignment level, when the accuracy and quality of the classification features are not affected, was determined as no greater than 50 µm. Obtained results led to development of image-processing algorithms for determination of the direction of misalignment and concurrent alignment of the bacterial colonies’ diffraction patterns. The proposed algorithms enable the rigorous monitoring and controlling of the measurement’s conditions in order to preserve the high accuracy of bacteria identification.

## 1. Introduction

The light diffraction on bacterial colonies has been extensively examined because of the promising potential of this method [1,2,3,4,5,6,7] against alternative destructive techniques based on mass spectroscopy [8,9,10], Raman spectroscopy [11,12,13], or phylogenetics [14,15]. The light is used as a carrier of specific information about the bacteria species/strains and can be retrieved and reproduced in a form of the unique optical signatures to enable simple, nondetrimental, and effective bacteria identification. The identification of bacteria strain/species is performed using classification models comparing the experimental diffraction pattern of the unknown bacteria with the reference diffraction patterns of a known bacteria species. The experiments already reported [3,4,16] have shown that the diffraction patterns are suitable sources of specific features (optical signatures), which can be extracted and used to build highly accurate classification models to distinguish particular bacteria strains/species. The proposed approach enables the identification of bacterial colonies with accuracy higher than 98% [5]. Similar results were also achieved by alternative methods based on forward scattering light patterns [1,2,3,17,18,19]. However, it is essential to identify and, in the end, eliminate any non-specific factors potentially having an impact on the accuracy of the identification.

Non-species/strain-dependent factors influencing the spatial distribution of diffraction patterns are critical aspects of a reliable bacteria identification process. Any random variability of features extracted from experimental patterns in relation to the reference features used in the classification model limits accuracy of bacteria identification. Therefore, all factors affecting the bacterial colonies’ diffraction patterns should be carefully examined. As previously reported in [20], positioning of the registration plane is one of the main factors influencing the light-focusing properties of bacterial colonies. Moreover, the type of the nutrient medium, time, and temperature of the incubation also affect the optical and morphological properties of bacteria colonies and, in consequence, also their Fresnel patterns [6,7,21,22,23,24]. Therefore, accurate bacteria identification requires not only the standardization of the incubation conditions, but also comparability of the recording conditions of diffraction patterns with similar features.

Any non-species-dependent variations of the classification features extracted from diffraction patterns may lead to the increase of the false positive and false negative results, which limits the classification accuracy. Therefore, in the current work the impact of the bacterial colony and illuminating beam misalignment on classification features extracted from colonies’ diffraction patterns was examined. The misalignment can be introduced by the sample scanning module and is associated with the minimal achievable movement increment of the used motorized X–Y translational stages and applied scanning and system centering protocols. It should also be taken into consideration with the other alternative methods based on the forward scattering patterns of bacterial colonies, particularly when the features extraction and classification are based on other approaches, such as the use of Zernike (GPZ) polynomials/moments [6,17,25], which are highly sensitive for any variations of optical signatures symmetry. According to our knowledge, the influence of this system-dependent factor on deformation of diffraction patterns has never been reported before. It was demonstrated that the relative misalignment or lateral dislocation of the bacterial colony and the illuminating beam significantly affect intensity distribution of diffraction patterns and, therefore, extracted features used in the classification process. Hence, it can be treated as a critical system-dependent factor limiting the identification accuracy. This paper proposed a novel algorithm to assure colony–light beam alignment. The algorithm was based on deformation of the quantitative features of diffraction patterns induced by misalignment. In studies presented here, the variation spectrum of extracted classification features and spatial distribution of diffraction patterns’ intensity used in classification models for bacteria identification were utilized as foundations for the misalignment correction algorithm. The examination was carried out on the *Escherichia coli* bacterial colonies and based on analysis of 1200 diffraction patterns. The quantitative features of diffraction patterns being the most sensitive to induced misalignment were determined. The minimal misalignment limiting the diffraction patterns’ intensity variation was identified. Based on these results, the image-processing guided algorithm for automatic colony–light beam alignment was proposed and verified. The proposed approach offers the determination of the misalignment direction (angle) with the relative error changing in a range from 0.71% to 7.14% and colony–light beam alignment based on monitoring features extracted from diffraction patterns. The algorithm is a suitable method of automatically monitoring and controlling the measurement’s conditions, enabling the highly accurate bacteria identification based on bacterial colonies’ diffraction patterns.

## 2. Materials and Methods

### 2.1. Bacteria Sample Preparation

The examination was carried out on colonies of *Escherichia coli* (ATCC 25922). The cultures were obtained from the microbiological laboratory of the Department of Epizootiology and Veterinary Administration with the Clinic of Infectious Diseases of the Wrocław University of Environmental and Life Science. Bacteria suspensions were incubated for 18 h at a temperature of 37 °C. Bacteria suspensions in 10^−5^ and 10^−6^ respective dilutions were seeded on the surface of the solid nutrient medium in Petri dishes with Columbia agar (Oxoid) to obtain 12–20 colonies per plate and incubated at 37 °C for 18 h. The colonies with a radius in the range from 0.6 to 0.8 mm were examined in the studies presented herein.

### 2.2. Optical System Configuration

**Figure 1:**
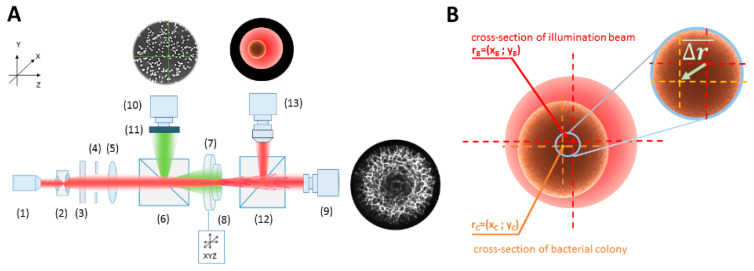
(**A**) The schematics of the miniaturized optical system for recording the diffraction patterns, where: (1) the laser diode module (635 nm, 1 mW, collimated, CPS635R, Thorlabs), (2) beam expander BE (1.5X, BE052-A, Thorlabs), (3) amplitude filters wheel (OD: 0–2, NDC-50C-2M-A, Thorlabs), (4) iris-diaphragm (diameter: 0–2.5 cm, ID25, Thorlabs), with automatically controlled diameter, (5) transforming lens L (f = 20 cm, AC254-200-A-ML, Thorlabs), (6) beam splitter (CM1-BP150, Thorlabs), (7) sample of bacterial colonies in the Petri dish on (8) automatic X–Y translation stage (travel range 150 mm, minimum incremental movement: 0.1 µm, NRT150/M, Thorlabs), (9) diffraction patterns recording CCD camera (EO-1312) with imaging objective (f = 3.5 cm, 59–872, Edmund Optics) and a computer unit (8). (**B**) The exemplary description of the analyzed ∆r-parameter of misalignment.

Examination of the *E. coli* bacteria was carried out using a newly designed, novel, miniaturized system for bacteria identification with converging spherical wave illumination [21] (see Figure 1A). The iris-diaphragm diameter and positioning of the Petri dish with colonies were controlled by the computer unit. Moreover, the additional measurement channel for image recording of all bacterial colonies on the Petri dish was included in the setup to enable the automatic localization of bacterial colonies grown on the medium. The channel combined the cage-mounted pellicle beam splitter (6), which enabled recording of bacterial colonies on the Petri dish in reflection mode, the additional (10) camera with imaging objective (f = 12 mm, Edmund Optics), and the ring illuminator for uniform illumination of the Petri dish. The second channel with a second cage-mounted pellicle beam splitter (12) and high-sensitivity CMOS camera (DCC1240M, Thorlabs) with imaging microscope objective (Nikon, 4x, WD = 30 mm) (13) was used to obtain the enlarged image of a single colony and determine the introduced colony–illuminating beam misalignment.

### 2.3. The Misalignment ∆r-Parameter Describing the Relative Dislocation of the Bacterial Colony and the Illuminating Beam’s Centers

The parameter ∆r was introduced as the misalignment of the center of the bacterial colony relative to the center of the fixed illuminating beam cross section (see Figure 1B), and it can be expressed by the following formula:

Δr = |√(x_C_ − x_B_)^2^ + (y_C_ − y_B_)^2^|, (1)

where (x_C_,y_C_) correspond to the coordinates of the center of the bacterial colony and (x_B_,y_B_) to the center of the illumination beam, which is the origin of the used coordinates system. The enlarged image of the individual colony registered in one of the measuring channels of the optical system was used for determination of the colony center by limiting the colony image by circle function. For this purpose a special ImageJ macro with human interaction to mark the diffraction pattern edges and center was written [26]. Next, the Δr-parameters were determined for different distances between the colony and illuminating beam centers (see Figure 1B). The measurements and analysis of variation of the colonies’ diffraction patterns were performed for six different misalignment Δr-parameters: 50, 100, 200, 300, 400, and 500 µm in four directions: vertical, horizontal, and two diagonally. The total number of diffraction patterns analyzed under this examination was equal to 1200.

### 2.4. Classification Features Extracted from Diffraction Patterns

For bacteria classification purposes, the dedicated image-processing and feature extraction algorithms developed by our group and described in detail in [3,4,18,24] were used. The classification features were extracted from 10 concentric annulus-shaped zones partitioning the diffraction patterns. For each of the zones, numerical features denoting morphological and textural properties based on the central statistical moments were calculated. The features included: mean value and standard deviation (sd) denoting brightness and roughness of the regions of interest, skewness referring to the measure of the symmetry of the shape of the pixel intensities distribution within each ring, kurtosis as a measure of the flatness of a distribution of the pixel intensities within each ring, smoothness, uniformity and entropy and the radius of the pattern. A total of 71 features for each diffraction pattern were denoted. The set of these features was used in our identification method for the building of classification models based on SVM (Support-Vector Machine) and examined with respect to the ∆r-parameter.

### 2.5. The Impact of the Misalignment ∆r-Parameter on the Variation of the Classification Features

ANOVA analysis at α = 0.05 was applied to assess statistical variation of the parameters affected by the ∆r-parameter and also to determine the minimal acceptable misalignment which did not affect accuracy of the bacteria identification. The *p*-value was used as a measure of the variation between the features extracted from the centered diffraction pattern and those recorded for various misalignments determined by the ∆r. The more variation of features indicated, the lower classification accuracy based on the diffraction pattern achieved. The centered patterns were used as reference diffraction signatures, allowing an accurate bacteria identification [5]. The analysis was performed on all 71 classification features at six values of the ∆r-parameter: 50, 100, 200, 300, 400, 500 µm. Then, the set of the features was limited to those that were the most efficient predictors of the bacteria species/strains.

### 2.6. The Sensitivity of Features Extracted form Diffraction Patterns on Misalignment ∆r-Parameter

**Figure 2:**
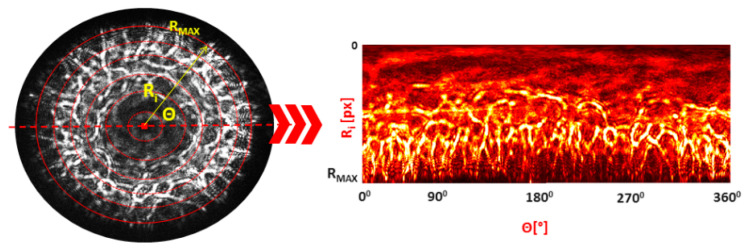
The schema of the proposed algorithm for the examination of the bacterial colony–illuminating beam misalignment influence on the spatial distribution of the Fresnel patterns (description in text).

The accuracy of the proposed identification method had its basis in an affinity of spatial distribution of diffracted light intensity within species and/or strains. Therefore, analysis of the sensitivity of diffraction patterns on introduced misalignments was an important part of improving classification procedure. The approach proposed here enabled quantitative analysis of the fluctuations in diffraction pattern intensity at different radial zones, leading to a determination of the regions where statistically significant variations of intensity values caused by introduced misalignments occurred. The sectional examination was used to determine the dependence of the diffraction pattern deformation relative to the direction of the misalignment, which enabled development of the automatic misalignment correction. To meet the challenges and enable correction procedures a novel dedicated image-processing algorithm was designed (see Figure 2).

**Figure 3:**
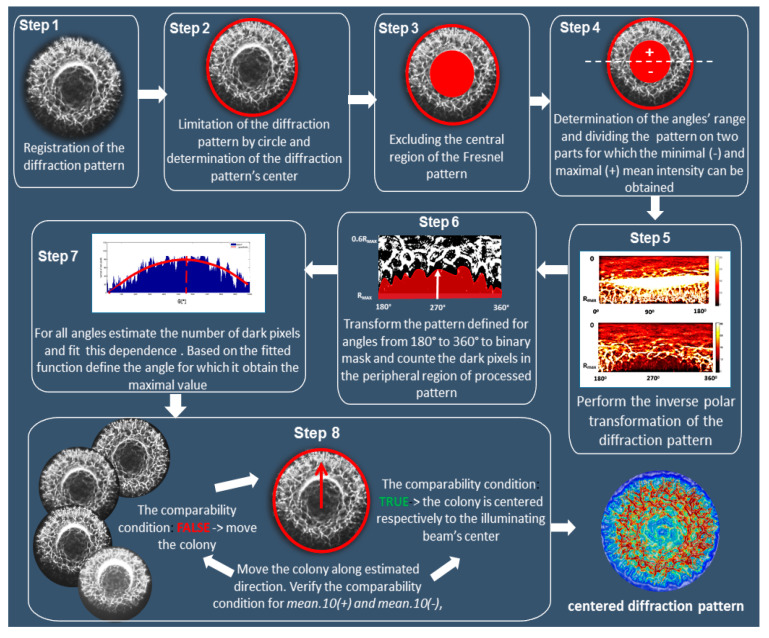
The concept of the image-guided algorithm for automatic positioning of bacterial colony relative to the center of the illuminating beam.

The algorithm was based on the inverse polar transformation of the circular diffraction pattern, which enabled precise examination of the variation of diffracted light intensity at radial distances <0; R_MAX_> at different Θ angles <0°;360°>. The resulting transformed pattern represented an interpolated diffraction pattern, where the circular structures were converted into rectangular. First, the diffraction pattern’s maximal radius R^MAX^ and origin (Ri = 0) were determined to locate the origin of the coordinate system, and the inverse polar transformation was carried out. After transformation, the set of new signatures, which represented redefined special distribution of intensity depending on R_i_ and Θ (see Figure 3), were obtained. It was also feasible to obtain new representations of the patterns at different values of the Θ angle: whole pattern at 0–360°; two parts with upper/lower at 0–180° and 180–360° and right/left at 90–270° and 270–90°; or quarters (0–90°, 90–180°, 180–270°, 270–360°). The processed patterns were limited by 10 rectangular-shaped partitioning zones with equal widths of 0.1 R_MAX_. For each of the zones the quantitative features indicated above were extracted and used for determination of the misalignment direction.

### 2.7. The Concept of an Algorithm for Automatic Detection and Correction of the Colony–Illuminating Beam Misalignment

Here we propose an image-processing method that can be used to determine the direction of the misalignment based upon the deformation of its diffraction pattern. However, it can be also applied to automatic aligning of the colony to the illuminating beam, to counteract the deformation and possible variation of the classification features.

The concept of this algorithm is presented in Figure 3 and described in the following steps:

**Step** **1:** Register the experimental diffraction pattern of a bacterial colony.
**Step** **2:** Limit the diffraction pattern by the shape function and determine its center.
**Step** **3:** Divide the pattern into 10 partitioning zones of equal widths. The number of limiting zones should be limited only to the peripheral ones. Based on the experimental measurement performed on colonies with a radius in the range 0.6–0.8 mm, this condition applies to the 6th to 10th zone.
**Step** **4:** Measure the mean intensity values of the 6th partitioning zone (mean.6) and determine the two parts of the diffraction patterns, with the highest and the lowest values of the mean.6. The most reliable indicator of the smallest misalignment is the mean.10 value. The Fresnel pattern is then divided into two parts with maximal (+) and minimal (−) mean intensity.
**Step** **5:** Perform the inverse polar transformation of (−) part of the pattern with the 1° angular resolution and divide into radial zones of the width 0.1 R_MAX_, where R_MAX_ is the radius of the circle limiting the diffraction pattern (determined in Step 2).
**Step** **6:** Transform the processed pattern including the peripheral (6–10) zones into a binary mask and determine the number of the dark pixels at each angle Θ (from 10th to 7th).
**Step** **7:** Derive the histogram representing the number of dark pixels at each Θ. Using the fitted dependence of the dark pixels’ number at specific angles, determine the misalignment angle based on the symmetry of the dependence.
**Step** **8:** Adjust positioning of the colony at the lowest possible resolution of the translation stage. Aligning conditions are considered with respect to the mean value of the 10th partitioning zone. For each dislocation the comparability condition relative to the values (mean.10(+) and mean.10(−)) of the mean.10 feature extracted from the peripheral zone of the (+) and (−) parts of decentered patterns is estimated. If mean.10(+) = mean.10(−), the colony and beam alignment is successfully achieved. If mean.10(+) ≠ mean.10(−), the dislocation should be continued until the intensity distribution of the diffraction pattern is symmetrical (mean.10(+) = mean.10(+)). When this condition is met, the colony and illuminating beam are aligned and the centered diffraction pattern can be registered.

## 3. Results and Discussions

### 3.1. The Dependence of Bacterial Colonies’ Diffraction Patterns on Misalignment

**Figure 4:**
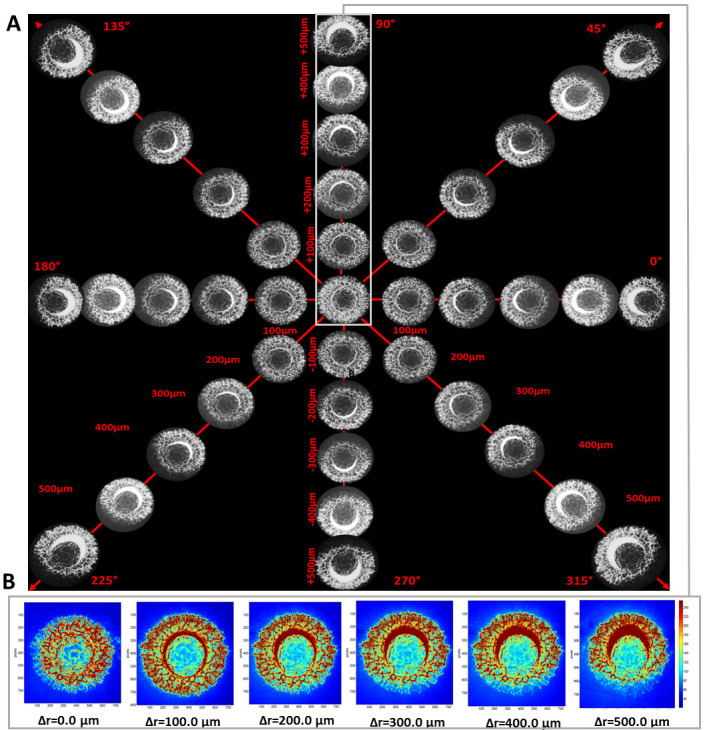
(**A**) The representative deformation of the diffraction patterns for various ∆r-parameter values (100–500 µm) along four different misalignment directions Θ = 0°;180°, 90°;270°, 135°;315°, 45°;225°. (**B**) The exemplary images of the bacterial colony diffraction patterns for different values of the ∆r-parameter in the vertical direction (Θ = 90°).

The deformation of the spatial intensity distribution induced by the misalignment was initially observed visually in the diffraction patterns. The exemplary patterns are presented in Figure 4A. The light intensity obtained for an ideally centered colony aligned with illuminating beam has circular, symmetric intensity distribution across the entire diffraction pattern. However, any deviations from the symmetry of the illumination, caused by dislocation of the colony relative to the beam center, led to the deformation of the pattern and its symmetry. The effect is shown in Figure 4B, where the changes of the discrete intensity values depending on the ∆r-parameter are given. With the increase of the ∆r-parameter, the intensity of the diffraction patterns exhibited non-symmetric spatial distribution and fluctuations in the intensity values.

**Figure 5:**
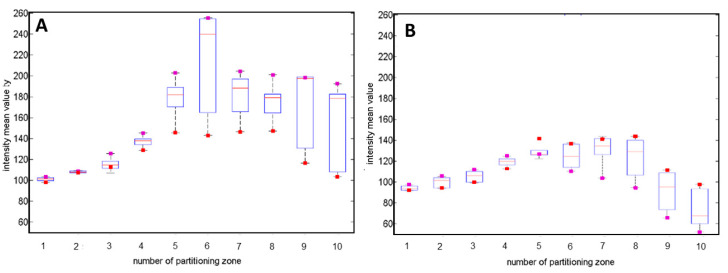
The representative boxplots demonstrating the variation of the diffraction patterns’ mean intensities across 10 partitioning zones of: (**A**) upper (for Θ = 0°–180°) and (**B**) lower (for Θ = 180°–360°) parts of the diffraction pattern (red square—mean intensity value of centered pattern for ∆r = 0 µm; violet square—mean intensity value of the decentered patterns for ∆r = +500 µm). Results obtained based on 200 diffraction patterns.

The direction of the deformations depended on the direction of colony displacement relative to the center of the illuminating beam. The preliminary analysis performed on 50 diffraction patterns was limited to the investigation of only one direction of the colony displacement. The deviations of the mean pixel intensity in different regions of the diffraction pattern along the vertical direction defined by Θ = 90° for the ∆r-parameter equal to 0 and 500 µm are presented in Figure 5. The intensity fluctuations were divided into 10 radial partitioning zones of equal width and examined, and are included in Figure 5. It was noted that the values of intensity at the upper part of the diffraction pattern (see Figure 5A) increased with the ∆r-parameter; however, the dynamics of the increase varied for different partitioning zones. Obtained results showed that the intensity variations in the central region (1–4 zones) were relatively small and the effect of the significant intensity fluctuations occurred at the peripheral regions (7–10 zones). In the upper part in the middle region (5–6 zones), the increase of the intensity was caused by the presence of the area of illuminating beam cross section, which was not overlapping the bacterial colony. Moreover, the intensity variations in the lower part (see Figure 5B) were smaller than in the upper part. The results indicated that the induced displacement of the colony in a specific direction led to a decrease of the light intensity in the peripheral region of the diffraction pattern in the corresponding direction (lower part), and to an increase of the intensity in the same region in the opposite direction (upper part). Therefore, this variation of the intensity can provide useful information for estimation of the colony dislocation direction relative to the center of illuminating beam, which is discussed in Section 3.3.

Sensitivity of the intensity variation in specific regions of the diffraction patterns to introduced misalignment was assessed by ANOVA. It was found that variations of the mean intensity of diffraction pattern partitioning zones caused by the ∆r-parameter were statistically significant (see Table 1).

The increase of the ∆r-parameter caused the increase in the number of the partitioning zones for which the statistically significant variation of mean intensity occurred (bolded values in Table 1). Moreover, the mean intensity from 6 and 10 partitioning zones can be used as the most sensitive indicator of the smallest misalignment of the illumination beam, and it can be applied for detection of the decentered diffraction patterns, which will be demonstrated in Section 3.3. Obtained results showed that system-dependent misalignment introduced statistically significant intensity variations. Therefore, it was important to take the misalignment into account for all alternative configurations of optical biosensors based on diffraction or forward-light scattering patterns [6,7,17,19]. It was particularly critical, when the features extraction and classification were carried out applying Chebyshev, Legendre, or Zernike moments [6,17,19], as they depend on the object’s symmetry [27,28]. As it was demonstrated above, any deviation of the optical signature’s symmetry caused by the misalignments, affected the classification accuracy.

### 3.2. The Quantitative Analysis of the Influence of the ∆r-Parameter on the Classification Features

The mean intensity, discussed in the previous section, is only one of the features used in the classification model for bacteria identification. Therefore, the influence of the misalignment on all classification features was examined for different ∆r-parameter values against the features extracted from the centered patterns. The results of the analysis performed on all 71 classification features extracted from 1200 recorded diffraction patterns at variable Δr-parameters is presented in Figure S1 in Supplementary Materials. With the increase of the ∆r-parameter, the comparability decreased for the features extracted from the peripheral regions of the patterns (zones 8–10) and for the middle region, when the illuminating beam did not overlap the colony (zone 6). On the other hand, the average values of classification features extracted from the partitioning zones near the central region (zones 1–3) of the diffraction patterns were comparable to the reference. This fully corresponded with the observations made and discussed in the previous section, where the changes of the mean intensity values from different partitioning zones were similar in nature. Obtained results showed that the most comparable classification feature values were achieved for the ∆r-parameter equal to 50 and 100 µm. The comparability of classification features at the ∆r-parameter greater than 100 µm was below 50%, and, therefore, impact on the correctness of the identification was significant (see Table 2).

Although the determination of proper features characterizing the unique spatial distribution of the patterns is critical for building effective predictive models, it is important to point out that not all of these analyzed features are good predictors. Therefore, apart from selecting the prediction model that provides the smallest possible error, there is an urge to select the features that enable differentiating bacteria most precisely. The ANOVA analysis of the subset of the classification features was used to narrow down the features to those exhibiting the highest classification potential. Comparability of the average values of these features had the highest impact on the accuracy of bacteria identification based on the diffraction patterns (see Figure S2 in Supplementary Materials). As in the previous case, the highest comparability of the classification features was obtained when the ∆r-parameter was equal to 50 and 100 µm. When the beam misalignment was at ∆r > 100 µm, the comparability of the features dropped, indicating a strong variation of the spatial distribution of the diffraction patterns’ intensity and, in consequence, a decrease of the classification accuracy. As observed in the above discussed results, the highest comparability was obtained for the features extracted from the partitioning zones located near the diffraction pattern’s center, since fluctuations of the pattern’s intensity in this region were relatively weak. For ∆r-parameter values greater than 200 µm the comparable features were located only in the central region of the patterns (1 partitioning zone). Results obtained for this subset of the classification features demonstrated that the comparability of the features slightly increased in comparison to the previous case (see Table 2). They indicated that the selection of the most predictive features may partially limit the influence of the misalignment. Obtained results also showed that for bacteria classification based on the colonies’ diffraction patterns, the limit value of the permissible misalignment is equal to 50 µm. Moreover, performed analysis demonstrated that the features mean.10 and mean.6 can be used as the main features, indicating the deformation of the diffraction patterns caused by the colony–illuminating beam misalignment is sensitive to even the smallest dislocation of the colony. However, it should be pointed out that the highest changes in the value of mean.6 were observed when the intensity increased in the part of the pattern opposite to the misalignment direction. Thus, it can be used to determine the direction of misalignment even for small values of the ∆r-parameter.

The impact of the misalignment was not analyzed for the alternative identification methods [6,7,17,19] and no attempts were made to quantitatively characterize its influence on classification accuracy. Some of these methods are based on the Zernike moments/polynomials [6,17,19], which are unique tools to capture information on circular patterns for feature extraction and classification. In this case, circular scattering patterns were downsized, centered, and the basic Zernike functions (being the complex radial polynomials exhibiting non-zero values inside of a united circle defined in polar coordinates) were evaluated. The basic function used as a symmetrical radial polynomial indicated that any deformation of the optical signature symmetry, as confirmed in this and previous sections, will cause the decrease of the Zernike moment values and, in consequence, will lead to a decrease of the identification accuracy by increasing false positive or false negative results of the classification. Therefore, the results presented here confirmed the impact of the deformation of the diffraction patterns on the accuracy of the classification process, and they should be taken into careful consideration in all alternative methods of bacteria identification based on optical patterns of bacterial colonies. Moreover, the potential misalignment of bacterial colony and illuminating beam should be treated as a significant system-dependent factor and corrected prior to the registration of the final optical patterns.

### 3.3. The Algorithm for Automatic Detection of the Colony–Illuminating Beam Misalignment and Correction of Diffraction Pattern Deformation

**Figure 6:**
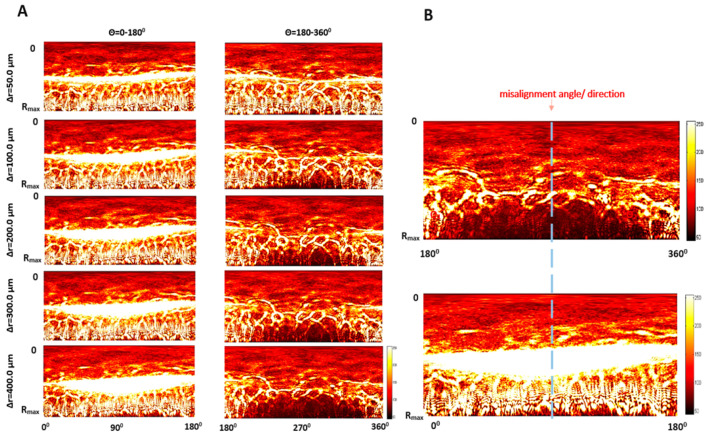
(**A**) The representative inverse polar images of the upper and lower parts of the diffraction patterns for different values of the ∆r-parameter along the vertical direction (Θ = 280°), (**B**) the concept of the use of diffraction pattern changes caused by the colony–illuminating beam misalignment for estimation of the misalignment direction for Θ = 180°–360°.

The results presented in Section 3.2 showed that a misalignment of bacterial colony to the illuminating beam equal to one-eighth of the colony radius (Δr = 100.0 µm) can cause over 12% variation in the classification features’ values, and misalignment equal to one-fourth of the colony radius will lead to a variation of nearly 54%. Therefore, as a reliable technical solution to eliminate this system-dependent factor we developed the image-processing algorithm discussed in the following sections. Before correction of the induced misalignment, it is necessary to determine its direction. The proposed image-processing algorithm for this purpose was based on the inverse polar transformation of diffraction patterns, and the resulting outcomes of applying the algorithm are presented in detail in Figure 6. In the case of the centered diffraction patterns (Δr = 0.0 µm), the intensity distribution had a circular symmetry. Induced misalignment at Θ = 280° led to the deviation of the intensity distribution (see Figure 6A).

In the middle region of the upper part (5–6 partitioning zones) the increase of the light intensity was observed. With the increase of the Δr-parameter value the intensity increased with the increasing number of the partitioning zones in the middle region of the diffraction pattern. Moreover, substantial changes were observed in the peripheral region, where the intensity of the radial spoke-like shaped maxima increased. For the upper part of the decentered pattern (opposite to the direction of the introduced misalignment) in the central region no significant intensity variations occurred.

In the lower part of the decentered pattern (direction corresponding to the misalignment), in the central region of the pattern, no significant intensity variation appeared. However, the deformation was noticed in middle and peripheral regions, where the increase of Δr caused the decrease of the intensity.

The regions with reduced intensity were located symmetrical to the direction of the illumination dislocation (see Figure 6B). It was associated with the circular symmetry of the diffraction patterns. For increasing Δr-parameter values the reduced intensity area expanded from the peripheral to central regions of the diffraction patterns. Therefore, it was possible to use the information about these changes to estimate the direction of the misalignment and use it to adjust positioning of the bacterial colony to achieve the symmetrical intensity spatial distribution. The above-described observations were used as the foundation for the proposed image-processing algorithm given in the Methodology section and discussed in the next paragraphs.

**Figure 7:**
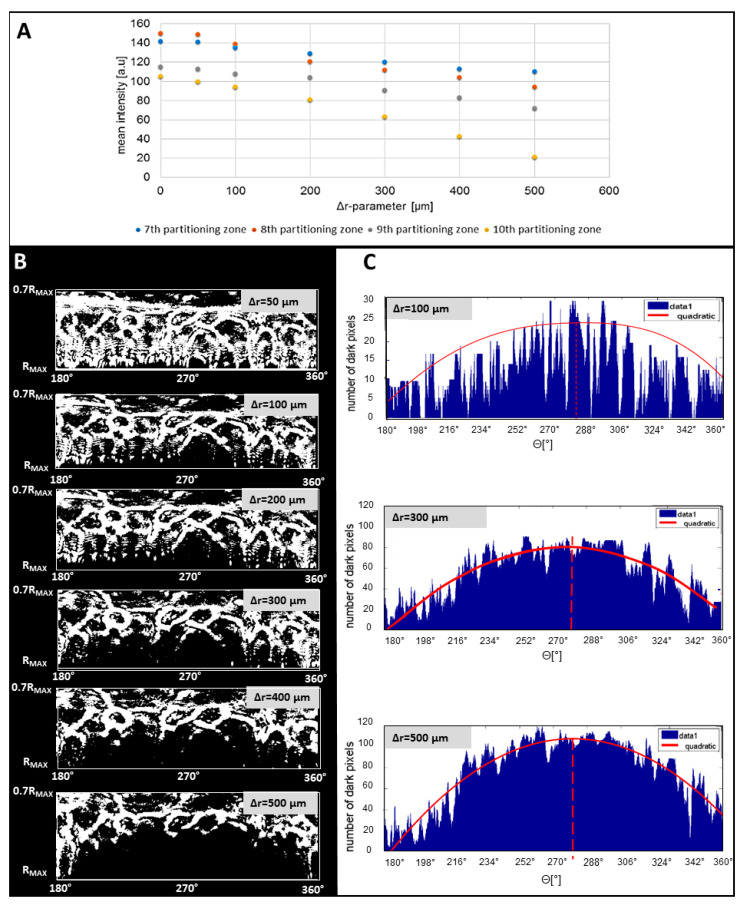
(**A**) Dependence of the mean intensity values from four peripheral partitioning zones on the Δr-parameter values; (**B**) The representative binary masks of the lower part of processed Fresnel patterns for different Δr-parameter values and for Θ = 280°; (**C**) The representative dependence of the number of dark pixels for each angle.

In the first step of applying the algorithm, the appropriate parts of the diffraction pattern (as upper part for Θ = 0°–180° and lower part for Θ = 180°–360° in the analyzed case) orthogonal to the direction of the misalignment were determined. This was achieved by control measurements and observation of the highest increase of mean intensity. The examinations discussed in Section 3.1 and Section 3.2 showed that in the lower part (Θ = 180°–360°) of the processed pattern zones 7th to 10th were the most relevant for such observation, as the mean intensity there strongly depended on the induced misalignment: Δr-parameter (see Figure 7A). As the area of the region of the decreased intensity increased for higher values of the Δr-parameter, it was therefore suitable for determination of the misalignment direction.

Next, the binary mask of the lower part of the processed pattern was obtained (see Figure 7B). The mask contained only the dark (0) and white (256) pixels. As it was proved, with the increase of the Δr-parameter, the area of the peripheral region of the processed pattern with the decreased intensity was larger. Therefore, the size of this area correlated to the Δr-parameter value. After binarization of the pattern, it was possible to evaluate its area by the count of the dark pixels from the pattern’s edges to the first intensity maximum. Considering the local variations of the intensity caused by speckles characteristic of the coherent light source, these maxima were experimentally defined as larger than 9 white pixels along the direction specified by the angle Θ. This step should be modified according to the imaging optics and pixel size of the camera used.

The number of dark pixels was counted at angles within the 180° to 360° range and the histogram was plotted (see Figure 7C). The histogram enabled to define the direction of the misalignment defined by angle Θ, through the maximum of the fitted histogram curve. The least-squares fit procedure was adapted, based on the coefficients of a second-order polynomial that fitted the obtained data, by minimizing the sum of the squares of the deviations of the model data. After fitting the second-order polynomial function (R^2^-statistic > 0.951, Adjusted-R^2^ > 0.942), it was possible to determine the maximal value of a specific value at the angle related to the direction of the misalignment. Depending on the angle, two parts of the diffraction patterns were defined orthogonal to the misalignment direction, determined by the occurrence of intensity increase and decrease (upper part at Θ = 0°–180° and lower part at Θ = 180°–360° in the presented example). In the data presented in Figure 7C, the dislocation of the colony was defined at Θ = 280°, and the angle determined through the fitted dependence of the number of the dark pixels on values was equal to 278°.

The accuracy of the proposed algorithm in the determination of the appropriate misalignment angles was examined and the results are presented in the Table 3. The highest relative error was equal to 7.14%; however, the region of dark pixels corresponding to the decreased intensity area was larger for higher values of the Δr-parameter. Therefore, additional examination of the erroneousness of misalignment angle determination for different Δr-parameter values was performed, and the results are presented in Table 4.

The results indicated that the performance of the proposed algorithm for determination of misalignment direction was limited for smaller values of the Δr-parameter. This effect was associated with the decreasing region of the diffraction pattern deformation for the smaller values of the Δr-parameter, which was indicated in previous sections (see Figures S1 and S2 in Supplementary Materials). The proposed algorithm enabled defining the appropriate positioning of the bacterial colony to achieve 100% comparability of features of the proposed classification model. Moreover, it eliminated the system-dependent misalignment causing the diffraction patterns’ deformation. The proposed approach can also be applied by the alternative methods of bacteria identification based on optical patterns of bacterial colonies, which use the Zernike moments for features extraction and classification process [1,6,17]. As it was indicated above, any deviation of optical pattern symmetry will cause the similarity comparison of decentered patterns with Zernike basic functions (radial, symmetrical polynomials) to decrease the Zernike moments and the classification accuracy. This problem can be eliminated by the proposed algorithm, enabling the registration of the centered patterns that can be used for further analysis based on Zernike moments.

The algorithm can be applied in analysis of single, separated bacteria colonies, of distinguishable edges from the background nutrient medium surface, because they are able to create diffraction patterns suitable for bacteria identification. In the case of the swarming colonies with a curled edge or margin, which are formed when the bacteria cells with high motility simultaneously grow and spread over a surface of nutrient medium, colonies often overlap or merge together. In consequence, the registered pattern is a superposition of the patterns of individual colonies rather than a single colony. These diffraction patterns of multiple colonies are affected by the spatial orientation of bacterial colonies, which is not suitable for bacteria identification purposes. The proposed algorithm of beam alignment would be affected by this random interference modulation of the diffraction patterns. However, by introducing the appropriate conditions of sample preparation as a limitation of nutrient surface wettability, it is possible to limit or even eliminate the bacterial cells’ motility and formation of the swarming or curled colonies. According to our knowledge, there is, so far, no other algorithm reported for centering the bacterial colony and illuminating beam based on analysis of diffraction patterns.

## 4. Conclusions

In this study, the influence of the relative bacterial colony–illuminating beam misalignment on the intensity spatial distribution of the bacterial colony diffraction patterns and the classification features was examined. This system-dependent factor affecting the identification accuracy is being reported for the first time. The misalignment of the bacterial colony and illuminating beam’s centers significantly affects the analyzed optical signatures, as well as extracted features used for bacteria classification. The mean intensities from the 6th and 10th partitioning zones of diffraction patterns are the most sensitive features, responding to even the smallest values of the introduced misalignment Δr-parameter. It was demonstrated that the most significant intensity fluctuations occurred in peripheral regions of the diffraction patterns, while in the center of the patterns they were relatively insignificant. Moreover, it was confirmed that the misalignment induced significant variations of the features extracted from the decentered patterns, compared to the centered one. Performed examination proved that the acceptable misalignment Δr-parameter for a microbiological diagnostics purpose should be not greater than 50 µm. Higher values of the Δr-parameter lead to the increase of the false positive and false negative results of the classification model. The misalignment is a crucial system-dependent factor that should be eliminated. Therefore, the proposed novel image-processing algorithm for determination of the misalignment direction and automatic adjustment or correction of the colony–illuminating beam centers enables determination of the misalignment’s angle with the error less than 8%. Moreover, the new concept of combining the proposed image-guided algorithm for misalignment direction and angle determination with the algorithm of monitoring the comparability of chosen features for appropriate bacterial colony alignment was proposed. It can be a tool for elimination of the diffraction patterns’ deformation and variation of the spatial distribution of the diffracted light intensity, leading to the undesirable variation of the classification features and affecting the classification accuracy. This approach can be easily adapted to alternative systems exploiting image recognition of optical patterns.

## Figures and Tables

**Table 1:** The results of ANOVA (numbers in bold represent statistically significant differences caused by the beam misalignment).

F-value	**Δr [µm]**	**Number of Partitioning Zone**									
		**1**	**2**	**3**	**4**	**5**	**6**	**7**	**8**	**9**	**10**
	50	0.09	0.01	0.12	0.04	0.55	**4.63**	2.02	0.66	0.21	**4.32**
	100	0.72	0.01	0.18	0.21	1.42	**4.42**	1.82	2.06	**4.51**	**11.18**
	200	0.20	3.46	3.52	**6.24**	**17.43**	**19.49**	**182.20**	**49.12**	**51.24**	**61.05**
	300	1.42	0.01	1.03	**4.45**	**19.55**	**24.90**	**193.11**	**52.39**	**60.21**	**72.22**
	400	0.01	0.02	1.66	**4.72**	**20.48**	**26.14**	**202.21**	**54.92**	**59.85**	**71.87**
	500	**0.43**	**4.43**	**4.52**	**4.95**	**14.55**	**24.90**	**193.11**	**52.39**	**7.34**	**4.91**
F-critical = 4.30											

**Table 2:** The percentage of classification features extracted from the decentered Fresnel patterns statistically comparable with the features of the centered Fresnel patterns for different sets of features.

**Set of All Classification Features**					
Δr = 50 µm	Δr = 100 µm	Δr = 200 µm	Δr = 300 µm	Δr = 400 µm	Δr = 500 µm
92.96%	76.06%	45.07%	28.17%	15.49%	7.04%
**Selected Subsets of the Classification Features**					
Δr = 50 µm	Δr = 100 µm	Δr = 200.µm	Δr = 300 µm	Δr = 400 µm	Δr = 500 µm
95.12%	87.80%	46.34%	36.59%	21.95%	9.76%

**Table 3:** The representative analysis of the proposed algorithm accuracy in the determination of the misalignment angles performed for 10 angles and 20 bacterial colonies for Δr = 200 µm.

Introduced Misalignment Angle Θ_0_	Estimated Mean Angle Θ_E_	ΔΘ *= |*Θ_0_−Θ_E_*|*	$\frac{\Delta \theta }{\theta_{0}}*100\%$
280°	278°	2°	0.71%
120°	122°	5°	4.16%
45°	40°	2°	4.44%
85°	89°	4°	4.71%
28°	32°	2°	7.14%
180°	185°	5°	2.78%
220°	119°	1°	0.45%
140°	144°	4°	2.86%
90°	86°	4°	4.44%
340°	335°	5°	1.47%

**Table 4:** The maximal percentage relative error (ΔΘ/Θ)·100% of the proposed algorithm accuracy in the determination of the misalignment angles performed for different Δr-parameter values.

Δr [µm]	$max\left(\frac{\Delta \theta }{\theta_{0}}*100\%\right)$
50	9.12%
100	8.21%
200	7.14%
300	6.54%
400	5.89%
500	5.45%

## Supplementary Materials

The following are available online at https://www.mdpi.com/1424-8220/20/20/5797/s1, Figure S1: The comparability of all classification features determined by *p*-values: blue color—no variation, yellow color—statistically significant variation (assumed criterion of comparability: *p*-value ≥ 0.05), Figure S2: The comparability of selected classification features’ subset determined by p-values: blue color—no variation, yellow color—statistically significant variation (assumed criterion of comparability: *p*-value ≥ 0.05).
