# Supplementary material for: Development of the Correction Algorithm to Limit the Deformation of Bacterial Colonies Diffraction Patterns Caused by Misalignment and Its Impact on the Bacteria Identification in the Proposed Optical Biosensor
Source: Sensors (Basel). 2020 Oct 13;20(20):5797. doi: 10.3390/s20205797 (PMC7602087; doi:10.3390/s20205797)
Supplement: Supplementary file 1 [file sensors-20-05797-s001.pdf]

sensors

MDPI

Supplementary Materials

Development of the Correction Algorithm to Limit the Deformation of Bacterial Colonies Diffraction Patterns Caused by Misalignment and Its Impact on the Bacteria Identification in the Proposed Optical Biosensor

Igor Buzalewicz [1,*], Agnieszka Suchwałko [2], Magdalena Karwańska [3], Alina Wieliczko [3] and Halina Podbielska [1]

[1] Bio-Optics Group, Department of Biomedical Engineering, Faculty of Fundamental Problems of Technology, Wrocław University of Science and Technology, 27 Wybrzeże S. Wyspiańskiego St., Wrocław, Poland; halina.podbielska@pwr.edu.pl

[2] QUANTUP, 30/9 Litewska St., Wrocław, Poland; agnieszka@quantup.pl

[3] Department of Epizootiology and Veterinary Administration with Clinic of Infectious Diseases, Faculty of Veterinary Medicine, Wrocław University of Environmental and Life Science, 45 Grunwaldzki Square, Wrocław, Poland; magdalena.karwanska@upwr.edu.pl (M.K.); alina.wieliczko@upwr.edu.pl (A.W.)

* Correspondence: igor.buzalewicz@pwr.edu.pl

Received: 24 August 2020; Accepted: 9 October 2020; Published: date

	Feature	Δr=50.0 µm p-value	Δr=100.0 µm p-value	Δr=200.0 µm p-value	Δr=300.0 µm p-value	Δr=400.0 µm p-value	Δr=500.0 µm p-value
10th zone	sd.10	0.0528	0.0006	0.0117	0.0412	0.0057	0.0081
	skewness.10	0.0480	0.0020	0.0015	0.0088	0.0128	0.0268
	flatness.10	0.1284	0.0021	0.0008	0.0048	0.0035	0.0021
	mean.10	0.0459	0.0498	0.0467	0.0441	0.0080	0.0012
	entropy.10	0.1025	0.0973	0.0495	0.0477	0.9840	0.0348
	uniformity.10	0.4523	0.3841	0.0366	0.0451	0.0258	0.0098
	smoothness.10	0.0421	0.0237	0.3167	0.0394	0.0125	0.0058
9th zone	sd.9	0.0598	0.0018	0.0001	0.0501	0.0354	0.0981
	skewness.9	0.2680	0.0004	0.0002	0.0006	0.0215	0.0001
	flatness.9	0.6540	0.0006	0.0002	0.0006	0.0004	0.0002
	mean.9	0.0559	0.1549	0.0164	0.7033	0.0958	0.0012
	entropy.9	0.7892	0.6526	0.1736	0.0919	0.0021	0.0005
	uniformity.9	0.0623	0.0632	0.0001	0.0005	0.0002	0.0001
	smoothness.9	0.0521	0.0441	0.0536	0.0454	0.0001	0.0001
8th zone	sd.8	0.8239	0.0522	0.0001	0.0004	0.0001	0.0001
	skewness.8	0.7865	0.6335	0.0017	0.0003	0.0025	0.0001
	flatness.8	0.0502	0.0128	0.9499	0.0002	0.0002	0.0001
	mean.8	0.8420	0.8012	0.0002	0.6746	0.0001	0.0001
	entropy.8	0.0782	0.0636	0.0004	0.0001	0.0004	0.0001
	uniformity.8	0.3215	0.2654	0.0722	0.0069	0.0026	0.0005
	smoothness.8	0.0789	0.0418	0.0019	0.0003	0.0012	0.0008
7th zone	sd.7	0.1982	0.1293	0.0322	0.0024	0.0382	0.0268
	skewness.7	0.3948	0.3142	0.0479	0.0064	0.1280	0.0328
	flatness.7	0.2988	0.2542	0.7554	0.0785	0.0482	0.0129
	mean.7	0.9002	0.8832	0.1028	0.0048	0.0026	0.0008
	entropy.7	0.0689	0.0566	0.0016	0.0001	0.0035	0.0009
	uniformity.7	0.2996	0.2435	0.0691	0.0011	0.0002	0.0001
	smoothness.7	0.6998	0.6422	0.7681	0.5550	0.0047	0.0012
6th zone	sd.6	0.3256	0.2569	0.0165	0.0051	0.0012	0.0006
	skewness.6	0.6580	0.5137	0.1298	0.0582	0.0012	0.0008
	flatness.6	0.0362	0.3139	0.0449	0.0129	0.0090	0.0032
	mean.6	0.0321	0.0968	0.0097	0.0185	0.0098	0.0042
	entropy.6	0.8623	0.8343	0.0270	0.0080	0.0012	0.0005
	uniformity.6	0.4211	0.3311	0.4394	0.3252	0.0079	0.0028
	smoothness.6	0.3268	0.2302	0.0054	0.0026	0.0018	0.0008
5th zone	sd.5	0.0512	0.0001	0.0002	0.0001	0.0001	0.0001
	skewness.5	0.0525	0.0080	0.0008	0.0001	0.0001	0.0001
	flatness.5	0.4215	0.0001	0.0001	0.0002	0.0001	0.0001
	mean.5	0.1239	0.0186	0.0002	0.0002	0.0001	0.0001
	entropy.5	0.8532	0.0041	0.0597	0.1608	0.0801	0.0105
	uniformity.5	0.3995	0.0496	0.2578	0.0437	0.0030	0.0009
	smoothness.5	0.0587	0.3537	0.0013	0.0004	0.0002	0.0001
4th zone	sd.4	0.3981	0.3564	0.0132	0.0053	0.0021	0.0009
	skewness.4	0.0519	0.8119	0.0736	0.0184	0.0099	0.0052
	flatness.4	0.0753	0.0652	0.0566	0.0174	0.0095	0.0005
	mean.4	0.3587	0.3290	0.0133	0.0004	0.0003	0.0001
	entropy.4	0.7025	0.6588	0.0011	0.0001	0.0001	0.0001
	uniformity.4	0.9862	0.9560	0.0142	0.0003	0.0002	0.0001
	smoothness.4	0.6102	0.5918	0.1012	0.0072	0.0038	0.0012
3th zone	sd.3	0.2188	0.1887	0.0434	0.0515	0.0101	0.0058
	skewness.3	0.3680	0.3068	0.9577	0.0478	0.0328	0.0250
	flatness.3	0.3698	0.3205	0.0388	0.0096	0.0482	0.0112
	mean.3	0.2159	0.1979	0.0563	0.0695	0.0558	0.0021
	entropy.3	0.3287	0.2794	0.0624	0.0017	0.0009	0.0005
	uniformity.3	0.6921	0.6111	0.0519	0.0517	0.0009	0.0002
	smoothness.3	0.8563	0.8367	0.0490	0.0326	0.0107	0.0085
2nd zone	sd.2	0.4210	0.3620	0.0624	0.0007	0.0005	0.0003
	skewness.2	0.8652	0.8343	0.3258	0.0563	0.0115	0.0098
	flatness.2	0.4215	0.3136	0.0134	0.0264	0.0198	0.0085
	mean.2	0.2153	0.1363	0.0520	0.0578	0.0524	0.0012
	entropy.2	0.2230	0.1257	0.0602	0.0501	0.0001	0.0001
	uniformity.2	0.1205	0.1052	0.0601	0.0002	0.0001	0.0001
	smoothness.2	0.4962	0.4028	0.0020	0.0005	0.0002	0.0001
1st zone	sd.1	0.4325	0.3448	0.0741	0.0546	0.0529	0.0509
	skewness.1	0.8921	0.8712	0.4104	0.0006	0.0004	0.0002
	flatness.1	0.4023	0.3651	0.0375	0.0001	0.0001	0.0001
	mean.1	0.6538	0.5373	0.1974	0.0603	0.0001	0.0001
	entropy.1	0.9658	0.9517	0.0779	0.0676	0.0601	0.0512
	uniformity.1	0.6801	0.6270	0.1722	0.7631	0.2164	0.1258
	smoothness.1	0.4128	0.3422	0.1658	0.1184	0.0958	0.0524
	radius	0.8024	0.6156	0.4334	0.1853	0.1121	0.0901

Figure S1. The comparability of all classification features determined by p-values: blue color - no variation, yellow color - statistically significant variation (assumed criterion of comparability: P-value≥0.05).

		Δr=50.0 μm	Δr=100.0 μm	Δr=200.0 μm	Δr=300.0 μm	Δr=400.0 μm	Δr=500.0 μm
	Feature	p-value	p-value	p-value	p-value	p-value	p-value
10th zone	sd.10	0.0528	0.0006	0.0117	0.0412	0.0057	0.0081
	mean.10	0.0459	0.0498	0.0487	0.0441	0.0080	0.0012
	entropy.10	0.1025	0.0973	0.0495	0.0477	0.9840	0.0348
	uniformity.10	0.4523	0.3841	0.0366	0.0451	0.0258	0.0098
9th zone	sd.9	0.0598	0.0018	0.0001	0.0001	0.0354	0.0981
	mean.9	0.0559	0.1549	0.0164	0.7033	0.0958	0.0012
	entropy.9	0.7892	0.6526	0.1736	0.0919	0.0021	0.0005
	uniformity.9	0.0723	0.0632	0.0001	0.0005	0.0002	0.0001
8th zone	sd.8	0.8239	0.0522	0.0001	0.0004	0.0001	0.0001
	mean.8	0.8420	0.8012	0.0002	0.6746	0.0001	0.0001
	entropy.8	0.0782	0.0636	0.0004	0.0001	0.0004	0.0001
	uniformity.8	0.3215	0.2654	0.0722	0.0069	0.0026	0.0005
7th zone	sd.7	0.1982	0.1293	0.0322	0.0024	0.0382	0.0268
	mean.7	0.9002	0.8832	0.1028	0.0048	0.0026	0.0008
	entropy.7	0.0689	0.0566	0.0016	0.0001	0.0035	0.0009
	uniformity.7	0.2996	0.2435	0.0691	0.0011	0.0002	0.0001
6th zone	sd.6	0.3256	0.2569	0.0165	0.0051	0.0012	0.0006
	mean.6	0.0321	0.0968	0.0097	0.0185	0.0098	0.0042
	entropy.6	0.8623	0.8343	0.0270	0.0080	0.0012	0.0005
	uniformity.6	0.4211	0.3311	0.4394	0.3252	0.0079	0.0028
5th zone	sd.5	0.0512	0.0001	0.0002	0.0001	0.0001	0.0001
	mean.5	0.1239	0.0386	0.0002	0.0002	0.0001	0.0001
	entropy.5	0.8532	0.8119	0.0597	0.1608	0.0801	0.0105
	uniformity.5	0.3995	0.3537	0.2578	0.0437	0.0030	0.0009
4th zone	sd.4	0.3981	0.3564	0.0132	0.0053	0.0021	0.0009
	mean.4	0.3587	0.3290	0.0133	0.0004	0.0003	0.0001
	entropy.4	0.7025	0.6588	0.0011	0.0001	0.0001	0.0001
	uniformity.4	0.9862	0.9560	0.0142	0.0003	0.0002	0.0001
3th zone	sd.3	0.2188	0.1887	0.0434	0.0515	0.0101	0.0058
	mean.3	0.2159	0.1979	0.0563	0.0695	0.0558	0.0021
	entropy.3	0.3287	0.2794	0.0624	0.0017	0.0009	0.0005
	uniformity.3	0.6921	0.6111	0.0519	0.0517	0.0009	0.0002
2nd zone	sd.2	0.4210	0.3620	0.0624	0.0007	0.0005	0.0003
	mean.2	0.2153	0.1363	0.0520	0.0578	0.0524	0.0012
	entropy.2	0.2230	0.1257	0.0602	0.0501	0.0001	0.0001
	uniformity.2	0.1205	0.1052	0.0601	0.0002	0.0001	0.0001
1st zone	sd.1	0.4325	0.3448	0.0741	0.0546	0.0529	0.0509
	mean.1	0.6538	0.5373	0.1974	0.0603	0.0001	0.0001
	entropy.1	0.9658	0.9517	0.0779	0.0676	0.0601	0.0512
	uniformity.1	0.6801	0.6270	0.1722	0.7631	0.2164	0.1258
	radius	0.8024	0.6156	0.4334	0.1853	0.1121	0.0901

Figure S2. The comparability of selected classification features' subset determined by p-values: blue color - no variation, yellow color - statistically significant variation (assumed criterion of comparability: P- value≥0.05).
